# Supplementary material for: Cancer-associated USP28 missense mutations disrupt 53BP1 interaction and p53 stabilization
Source: Nat Commun. 2025 Dec 9;16:10310. doi: 10.1038/s41467-025-66341-3 (PMC12689633; doi:10.1038/s41467-025-66341-3)
Supplement: Supplementary file 1 — Supplementary Information [file 41467_2025_66341_MOESM1_ESM.pdf]

Supplementary Information

Cancer-Associated USP28 Missense Mutations Disrupt 53BP1 Interaction and p53 Stabilization

Authors: Hazrat Belal[1], Esther Feng Ying Ng[1], Midori Ohta[1], Franz Meitinger[1]*

Affiliations:
[1]Okinawa Institute of Science and Technology Graduate University, Okinawa 904-0495, Japan.

*Corresponding author: franz.meitinger@oist.jp

Running Head: USP28 in mitotic stress response

Figure S1

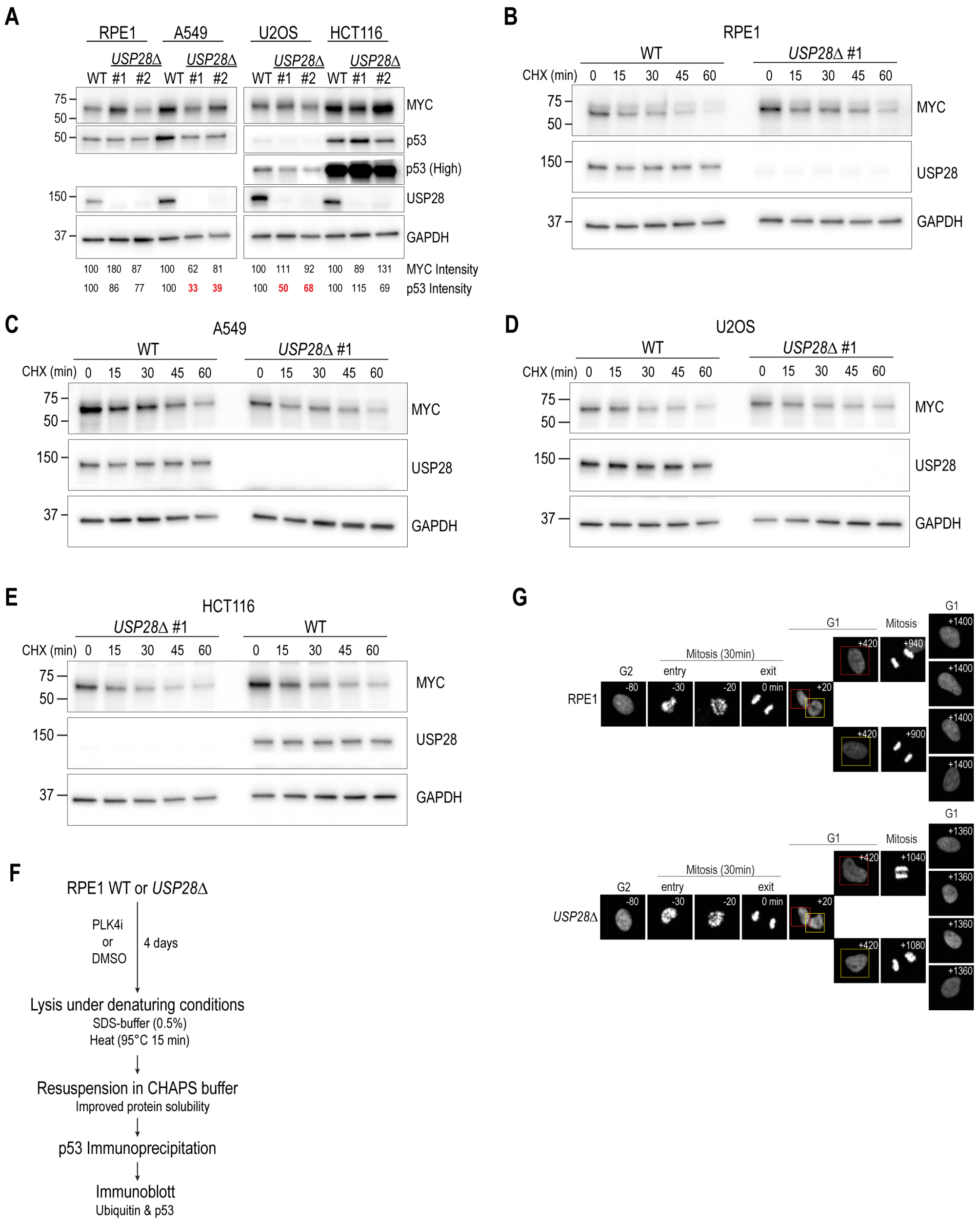

A
RPE1
A549
U2OS
HCT116
USP28Δ
WT #1 #2 WT #1 #2 WT #1 #2 WT #1 #2
75
50
150
37
MYC
p53
p53 (High)
USP28
GAPDH
100 180 87 100 62 81 100 111 92 100 89 131 MYC Intensity
100 86 77 100 33 39 100 50 68 100 115 69 p53 Intensity
B
RPE1
WT
USP28Δ #1
CHX (min) 0 15 30 45 60 0 15 30 45 60
MYC
USP28
GAPDH
C
A549
WT
USP28Δ #1
CHX (min) 0 15 30 45 60 0 15 30 45 60
MYC
USP28
GAPDH
D
U2OS
WT
USP28Δ #1
CHX (min) 0 15 30 45 60 0 15 30 45 60
MYC
USP28
GAPDH
E
HCT116
USP28Δ #1
WT
CHX (min) 0 15 30 45 60 0 15 30 45 60
MYC
USP28
GAPDH
F
RPE1 WT or USP28Δ
PLK4i or DMSO
4 days
Lysis under denaturing conditions
SDS-buffer (0.5%)
Heat (95°C 15 min)
Resuspension in CHAPS buffer
Improved protein solubility
p53 Immunoprecipitation
Immunoblott
Ubiquitin & p53
G
RPE1
G2
Mitosis (30min)
entry
exit
G1
Mitosis
G1
-80
-30
-20
0 min
+20
+420
+940
+900
+1400
USP28Δ
+1040
+1080
+1360

Figure S1: Effect of USP28 deletion on p53 and MYC stability.

(A) Analysis of p53 and MYC expression in cell lines with the indicated geneotypes. Red numbers indicate samples in which p53 was consistently downregulated in two independent clones. GAPDH served as a loading control. The samples derive from the same experiment, but different gels for USP28, MYC, GAPDH, and another for p53 were processed in parallel. Representative immunoblots from two independent experiments with similar results.

(B-F) Analysis of MYC stability in control and USP28 deleted clones following cycloheximide treatment in RPE1 (B), A549 (C), U2OS (D) and HCT116 (E) cells. GAPDH served as loading control. The samples derive from the same experiment and were processed on the same gel. Representative immunoblots from two independent experiments with similar results.

(F) Schematic of the denaturing immunoprecipitation assay used to assess USP28-dependent ubiquitination of p53, as shown in Figure 1G.

(G) Imaging-based assay evaluating daughter cell fate after prolonged mitosis in wildtype and *USP28*Δ RPE1 cells (Related to Fig. 2A). Cells underwent transient mitotic arrest via Monastrol, followed by washout and imaging for 48 h. Representative examples of cells with 30 min mitotic duration are shown. Scale bar: 10 µm.

Figure S2

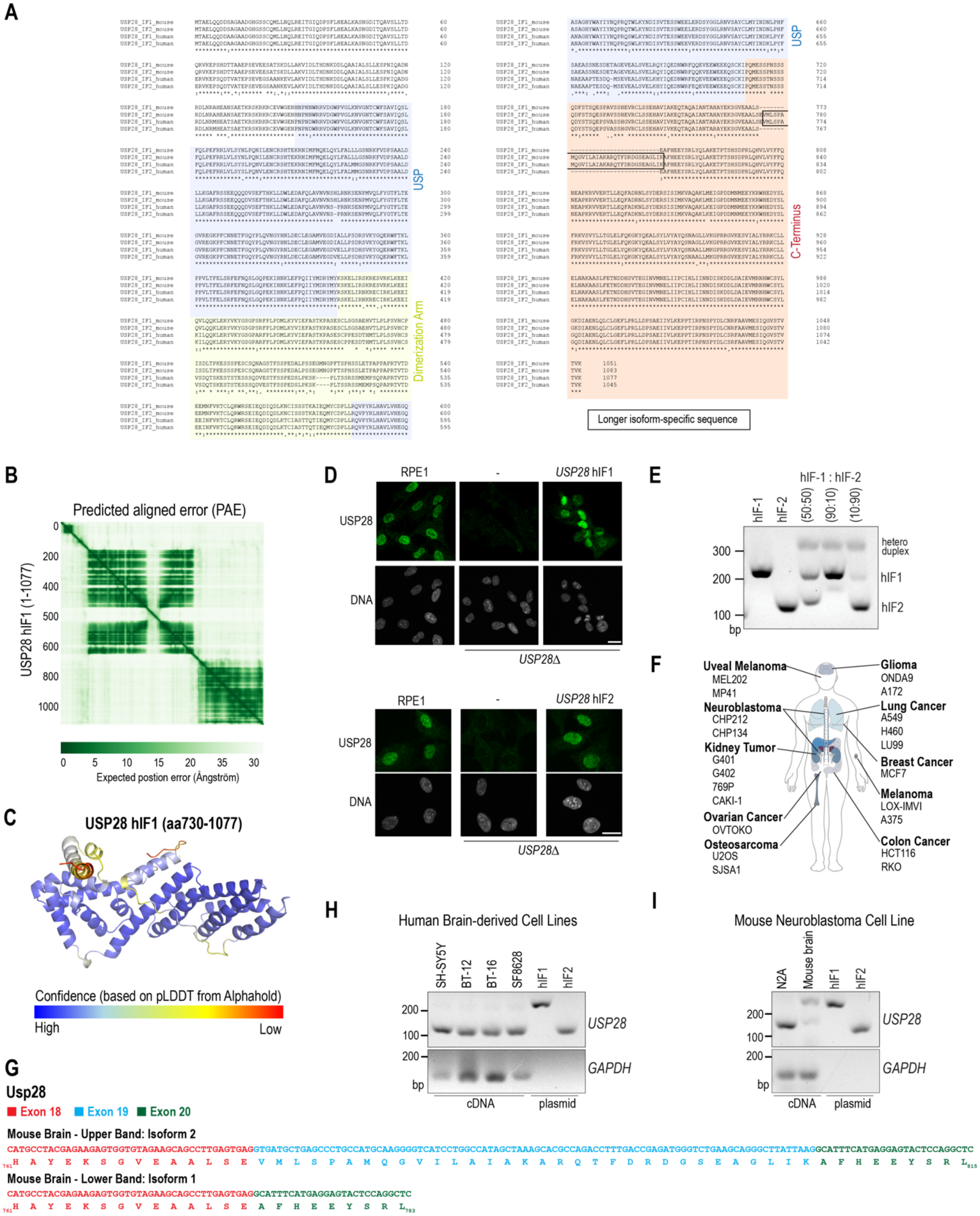
A
USP
Dimerization Arm
C-Terminus
Longer isoform-specific sequence
B
Predicted aligned error (PAE)
USP28 hIF1 (1-1077)
Expected postion error (Ångström)
C
USP28 hIF1 (aa730-1077)
Confidence (based on pLDDT from Alphahold)
High
Low
D
RPE1
USP28 hIF1
USP28 hIF2
USP28
DNA
USP28Δ
E
hIF-1 : hIF-2
hIF-1
hIF-2
(50:50)
(90:10)
(10:90)
hetero duplex
hIF1
hIF2
bp
F
Uveal Melanoma
MEL202
MP41
Neuroblastoma
CHP212
CHP134
Kidney Tumor
G401
G402
769P
CAKI-1
Ovarian Cancer
OVTOKO
Osteosarcoma
U2OS
SJSA1
Glioma
ONDA9
A172
Lung Cancer
A549
H460
LU99
Breast Cancer
MCF7
Melanoma
LOX-IMVI
A375
Colon Cancer
HCT116
RKO
H
Human Brain-derived Cell Lines
SH-SY5Y
BT-12
BT-16
SF8628
hIF1
hIF2
USP28
GAPDH
cDNA
plasmid
I
Mouse Neuroblastoma Cell Line
N2A
Mouse brain
G
Usp28
Exon 18
Exon 19
Exon 20
Mouse Brain - Upper Band: Isoform 2
CATGCCTACGAGAAGAGTGGTGTAGAAGCAGCCTTGAGTGAGGTGATGCTGAGCCCTGCCATGCAAGGGGTCATCCTGGCCATAGCTAAAGCACGCCAGACCTTTGACCGAGATGGGTCTGAAGCAGGGCTTATTAAGGCATTTCATGAGGAGTACTCCAGGCTC
761 H A Y E K S G V E A A L S E V M L S P A M Q G V I L A I A K A R Q T F D R D G S E A G L I K A F H E E Y S R L 815
Mouse Brain - Lower Band: Isoform 1
CATGCCTACGAGAAGAGTGGTGTAGAAGCAGCCTTGAGTGAGGCATTTCATGAGGAGTACTCCAGGCTC
761 H A Y E K S G V E A A L S E A F H E E Y S R L 783

Figure S2: Comparison of long and short isoforms of USP28 in human and mice.

(A) Sequence alignment of the two primary USP28 isoforms in human and mice, highlighting conserved regions. See text for more explanation.

(B) AlphaFold-predicted aligned error map for the full-length USP28^{IF1} structure, indicating regions of higher and lower confidence in the structural model.

(C) Confidence score of the AlphaFold model for the full-length USP28^{IF1} structure.

(D) Representative immunostaining images of USP28, showing nuclear localization of endogenous USP28 and transgene-expressed *USP28*^{hIF1} and *USP28*^{hIF2} (clonal cell lines). The nucleus was visualized with the DNA-staining compound Hoechst 33342. Images on the right are also shown in Figure 6B. Scale bar: 10 µm.

(E) PCR-based assay to assess sensitivity in mixtures containing varying ratios of USP28 hIF1 and hIF2 plasmids.

(F) Overview of cancer-specific cell lines shown in Figure 2H.

(G) Nucleotide sequences of the expressed *USP28* mRNA confirming that isoforms with and without exon 19 are expressed in the mouse brain.

(H-I) RT-PCR analysis revealing that cancer-derived cell lines from human (H) and mouse (I) predominantly express the shorter isoforms *USP28*^{hIF2} and *USP28*^{mIF1}. Plasmids containing *USP28*^{hIF1} or *USP28*^{hIF2} are used as control. A mouse brain sample is used for comparison (I). Representative of two independent repeats.

Figure S3

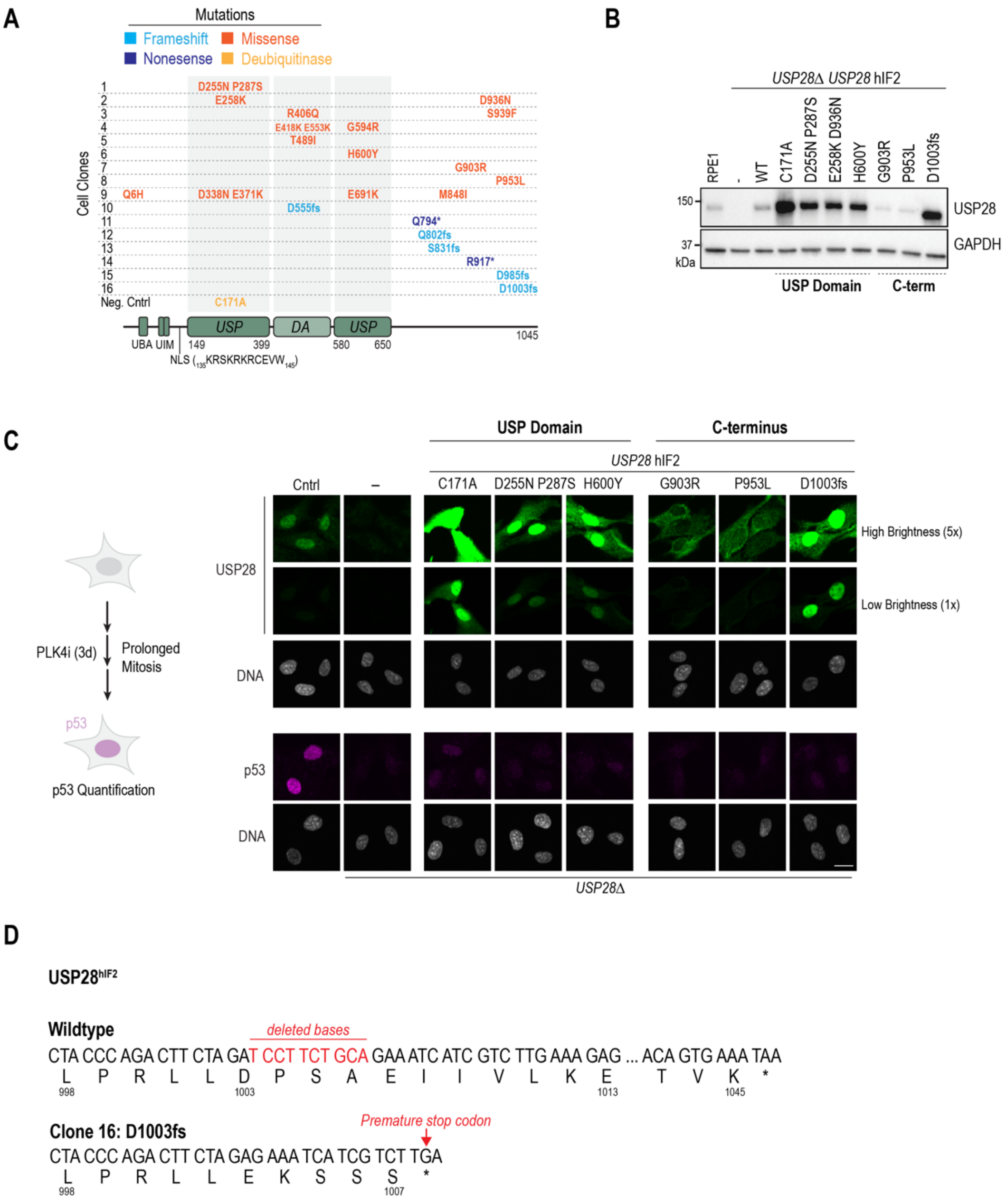
A
Mutations
Frameshift
Missense
Nonesense
Deubiquitinase
Cell Clones
D255N P287S
E258K
R406Q
E418K E553K
G594R
T489I
H600Y
D936N
S939F
G903R
P953L
Q6H
D338N E371K
E691K
M848I
D555fs
Q794*
Q802fs
S831fs
R917*
D985fs
D1003fs
Neg. Cntrl
C171A
USP
DA
USP
UBA UIM
149
399
580
650
1045
B
USP28Δ USP28 hIF2
RPE1
-
WT
C171A
D255N P287S
E258K D936N
H600Y
G903R
P953L
D1003fs
150
37
kDa
USP28
GAPDH
USP Domain
C-term
C
USP Domain
C-terminus
USP28 hIF2
Cntrl
–
C171A
D255N P287S
H600Y
G903R
P953L
D1003fs
USP28
High Brightness (5x)
Low Brightness (1x)
DNA
p53
DNA
PLK4i (3d)
Prolonged Mitosis
p53
p53 Quantification
USP28Δ
D
USP28hIF2
Wildtype
deleted bases
CTA CCC AGA CTT CTA GAT CCT TCT GCA GAA ATC ATC GTC TTG AAA GAG ... ACA GTG AAA TAA
L P R L L D P S A E I I V L K E T V K *
998
1003
1013
1045
Clone 16: D1003fs
Premature stop codon
CTA CCC AGA CTT CTA GAG AAA TCA TCG TCT TGA
L P R L L E K S S S *
998
1007

Figure S3: Characterization of spontaneous mutations in USP28

(A) Cell clones expressing *USP28*^{hIF2} with spontaneous mutations. The locations of clone-specific mutations within the USP28 gene are illustrated. The generation of these clones is depicted in Figure 3A. A summary is shown in Figure 3B.

(B) Cell lysates of mitotically arrested cells (Nocodazole, 100 ng/ml, 16 h) with the indicated genotype. The expression of wildtype and mutant USP28 is shown for comparison. GAPDH served as a loading control. The samples derive from the same experiment and were processed on the same gel. Representative immunoblots from two independent experiments with similar results.

(C) Visualization of the USP28 expression and localization in control, *USP28*Δ and *USP28*^{hIF2} mutant transgene expressing *USP28*Δ RPE1 cells (see Figure 3D for comparison). Visualization of p53 expression and stability following three days of PLK4 inhibition to prolong mitosis as indicated in the left schematic. The nucleus was visualized with the DNA-staining compound Hoechst 33342. Quantification of nuclear USP28 levels is shown in Figure 3D. Scale bar: 10 µm.

(D) Illustration of the genetic alteration of clone 16 (D1003fs), which has a 10-base deletion at codon 1003 leading to a premature stop codon at position 1008.

Figure S4

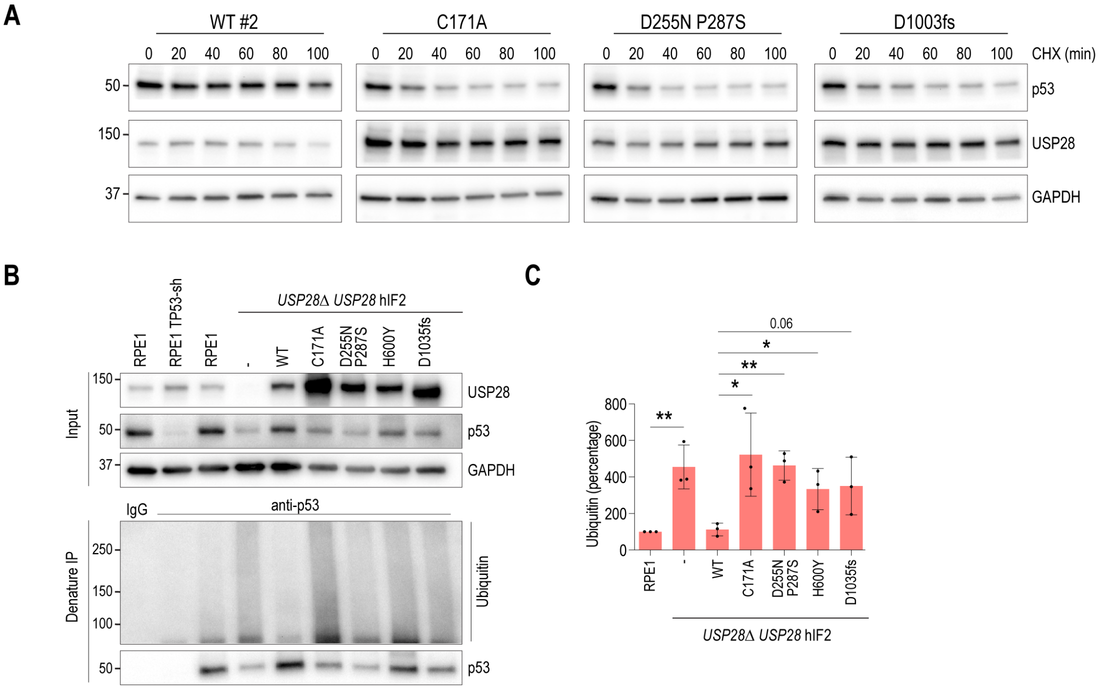
A
WT #2
C171A
D255N P287S
D1003fs
0 20 40 60 80 100
CHX (min)
p53
USP28
GAPDH
50
150
37
B
RPE1
RPE1 TP53-sh
RPE1
USP28Δ USP28 hIF2
-
WT
C171A
D255N P287S
H600Y
D1035fs
Input
Denature IP
IgG
anti-p53
Ubiquitin
250
100
C
Ubiquitin (percentage)
0
200
400
600
800
0.06
*
**
USP28Δ USP28 hIF2

Figure S4: Analysis of p53 stability in cells expressing wildtype and mutant USP28 transgenes

(A) Cycloheximide chase assays to assess p53 half-life in RPE1 *USP28*Δ cells expressing either wildtype or mutant *USP28*^{IF2} transgenes. Cells were treated with DMSO or PLK4 inhibitor (PLK4i) for 4 days prior to the assay. GAPDH, loading control. The samples derive from the same experiment and were processed on the same gel. Representative immunoblots from three independent experiments with similar results.

(B) Ubiquitination assay in RPE1 WT, TP53-sh (p53-depleted) and *USP28*Δ cells expressing wildtype and mutant transgenes after four days of PLK4 inhibition. GAPDH served as a loading control. IP, immunoprecipitation. Input, soluble supernatant. The samples derive from the same experiment but different gels for USP28, p53, GAPDH, and another for Ubiquitin were processed in parallel. Representative immunoblots from two independent experiments with similar results.

(C) Quantification of three independent experiments. Values for RPE1 and *USP28*Δ are also included in Figure 1G. Data are presented as mean ± SD. Statistical significance was determined using Student's t-test (RPE1 vs. USP28Δ, P = 0.0070, CI (95%) = 161.8 to 547.8; WT vs. C171A, P = 0.0368, CI (95%) = 40.81 to 779.0; WT vs. D255N P287S, P = 0.0023, CI (95%) = 209.4 to 491.4; WT vs. H600Y, P = 0.0315, CI (95%) = 31.95 to 410.7; WT vs. D1035fs, P = 0.0626, CI (95%) = -20.12 to 496.9).

Figure S5

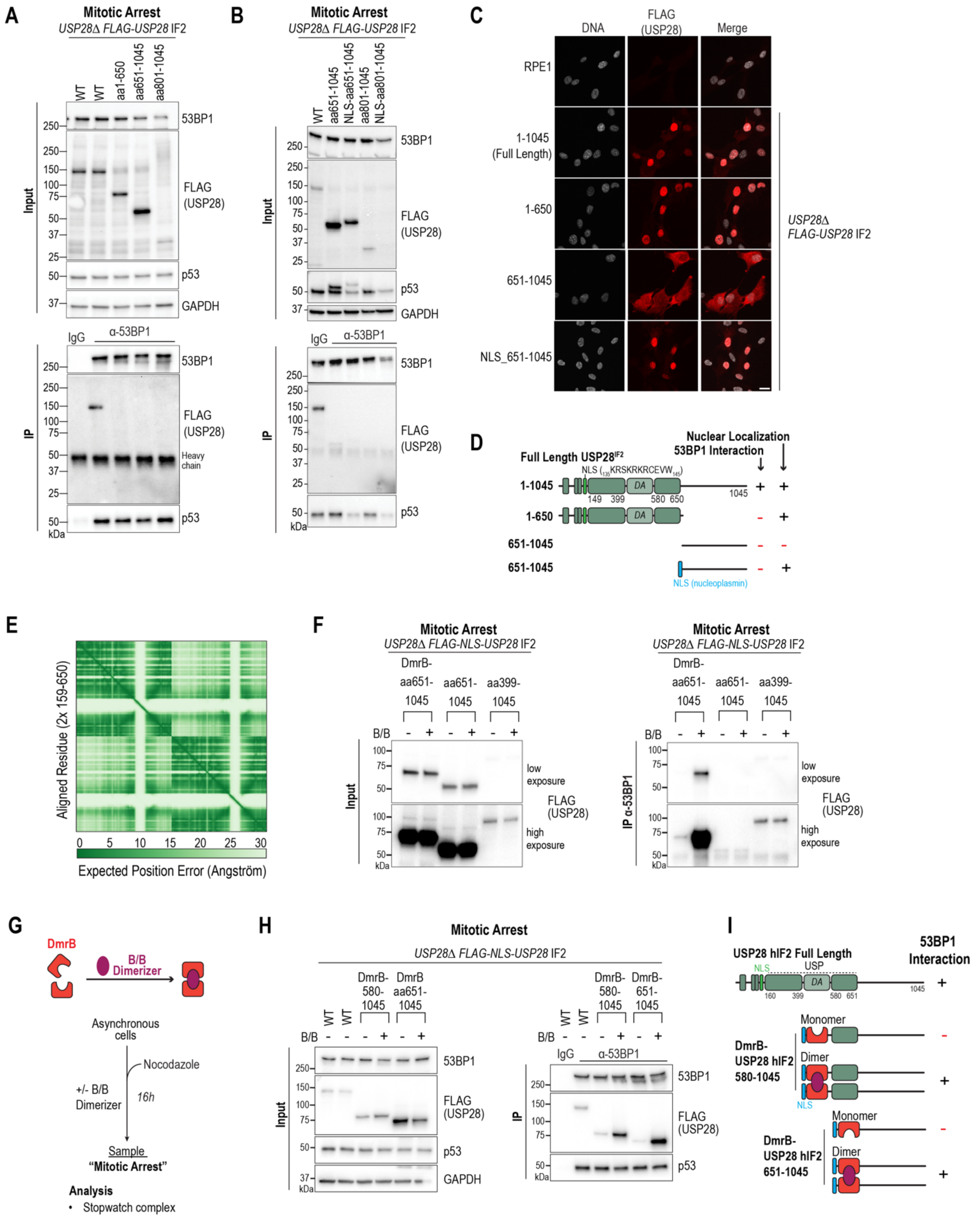

A
Mitotic Arrest
USP28Δ FLAG-USP28 IF2
53BP1
FLAG (USP28)
p53
GAPDH
Input
IP
α-53BP1
Heavy chain
B
C
DNA
FLAG (USP28)
Merge
RPE1
1-1045 (Full Length)
1-650
651-1045
NLS_651-1045
USP28Δ FLAG-USP28 IF2
D
Full Length USP28
Nuclear Localization
53BP1 Interaction
NLS (nucleoplasmin)
E
Aligned Residue (2x 159-650)
Expected Position Error (Angström)
F
USP28Δ FLAG-NLS-USP28 IF2
low exposure
high exposure
IP α-53BP1
G
DmrB
B/B Dimerizer
Asynchronous cells
Nocodazole
+/- B/B Dimerizer
16h
Sample
"Mitotic Arrest"
Analysis
Stopwatch complex
H
I
USP28 hIF2 Full Length
53BP1 Interaction
Monomer
Dimer
DmrB-USP28 hIF2 580-1045
DmrB-USP28 hIF2 651-1045

Figure S5: Characterization of USP28 domains that are crucial for the interaction with 53BP1

(A-B) Analysis of 53BP1 immunoprecipitates to determine complex formation with USP28 fragments in mitotically arrested cells (Nocodazole, 100 ng/ml, 16 h). The USP28 fragment 801-1045 was not stably expressed. (B) The USP28 fragment 651-1045 was fused to an NLS from nucleoplasmin. GAPDH served as a loading control. IP, immunoprecipitation. Input, soluble supernatant. The samples derive from the same experiment but different gels for 53BP1, p53, GAPDH, and another for Flag-USP28 were processed in parallel. Representative immunoblots from two independent experiments with similar results.

(C) Subcellular localization of USP28 fragments with and without the NLS, showing the influence of NLS on nuclear targeting. Scale bar: 10 µm.

(D) Overview of tested USP28 fragments in (A-C) and their capacity to localize in the nucleus and interact with 53BP1 in mitotically arrested cells. Endogenous and nucleoplasmin NLS are shown in green and blue, respectively.

(E) AlphaFold-predicted aligned error map for the USP28 dimer structure, indicating regions of varying confidence in the predicted structural model.

(F) Immunoblot showing expression levels of USP28 fragments under different exposure conditions to illustrate relative abundance. The USP28 blotting data from the same membrane with two different exposure times is shown in Figure 4I.

(G) Schematic of an inducible dimerization domain. Asynchronous cells were treated for 16h with Nocodazole (100 ng/ml) and the B/B dimerizer (100 nM) to induce dimerization. Mitotic cells were harvested and analyzed by immunoprecipitation.

(H) Immunoprecipitation analysis showing that USP28 C-terminal fragments (amino acids 580-1045 and 651-1045) form a complex with 53BP1 as a dimer induced by DmrB in the presence of B/B Dimerizer but not as a monomer in the absence of the B/B Dimerizer, indicating dimerization-dependent interaction. GAPDH served as a loading control. IP, immunoprecipitation. Input, soluble supernatant. The samples derive from the same experiment and were processed on the same gel. Representative immunoblots from two independent experiments with similar results. See also Figure 4H-J.

(I) Summary of (H) illustrating the requirement of USP28 dimerization for effective C-terminal interaction with 53BP1.

Figure S6

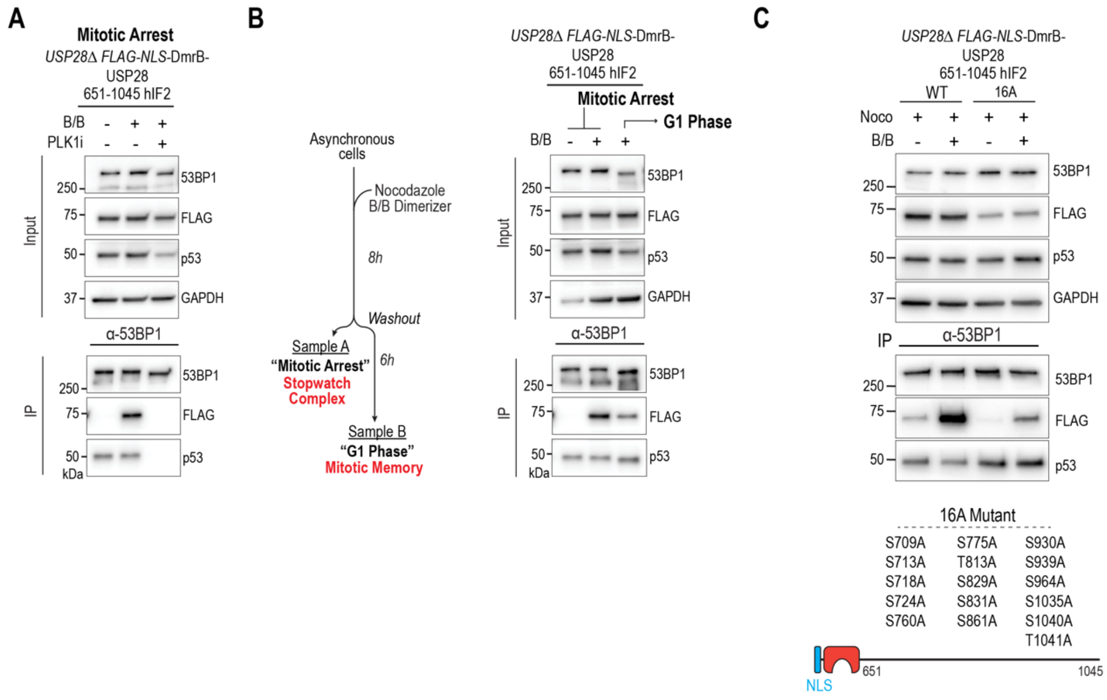
A
Mitotic Arrest
USP28Δ FLAG-NLS-DmrB-USP28 651-1045 hIF2
B/B - + +
PLK1i - - +
Input
IP
α-53BP1
53BP1
FLAG
p53
GAPDH
250
75
50
37
kDa
B
Asynchronous cells
Nocodazole B/B Dimerizer
8h
Washout
6h
Sample A
"Mitotic Arrest"
Stopwatch Complex
Sample B
"G1 Phase"
Mitotic Memory
USP28Δ FLAG-NLS-DmrB-USP28 651-1045 hIF2
Mitotic Arrest
G1 Phase
B/B - + +
C
USP28Δ FLAG-NLS-DmrB-USP28 651-1045 hIF2
WT
16A
Noco + + + +
B/B - + - +
16A Mutant
S709A
S713A
S718A
S724A
S760A
S775A
T813A
S829A
S831A
S861A
S930A
S939A
S964A
S1035A
S1040A
T1041A
NLS
651
1045

Figure S6: The stable interaction between the C-terminus of USP28^{hIF2} and 53BP1 mediates mitotic memory

(A) Immunoprecipitation analysis of 53BP1 in cells expressing a synthetic dimerized USP28 C-terminal construct, performed with or without PLK1 inhibition. GAPDH served as a loading control. IP, immunoprecipitation. Input, soluble supernatant. The samples derive from the same experiment and were processed on the same gel. Representative immunoblots from two independent experiments with similar results.

(B) Immunoprecipitation analysis of 53BP1 after release from mitotic arrest into G1 phase, used to evaluate the stability of its interaction with the synthetic dimerized USP28 C-terminus. GAPDH served as a loading control. IP, immunoprecipitation. Input, soluble supernatant. The samples derive from the same experiment and were processed on the same gel. Representative immunoblots from two independent experiments with similar results.

(C) Immunoprecipitation analysis of 53BP1 in cells expressing synthetic dimerized USP28 C-terminal constructs. The wildtype (WT) serves as a control, and the phosphorylation-deficient mutant (16A) carries alanine substitutions at 16 predicted PLK1 phosphorylation sites. GAPDH served as a loading control. IP, immunoprecipitation. Input, soluble supernatant. The samples derive from the same experiment and were processed on the same gel. Representative immunoblots from two independent experiments with similar results.

Figure S7

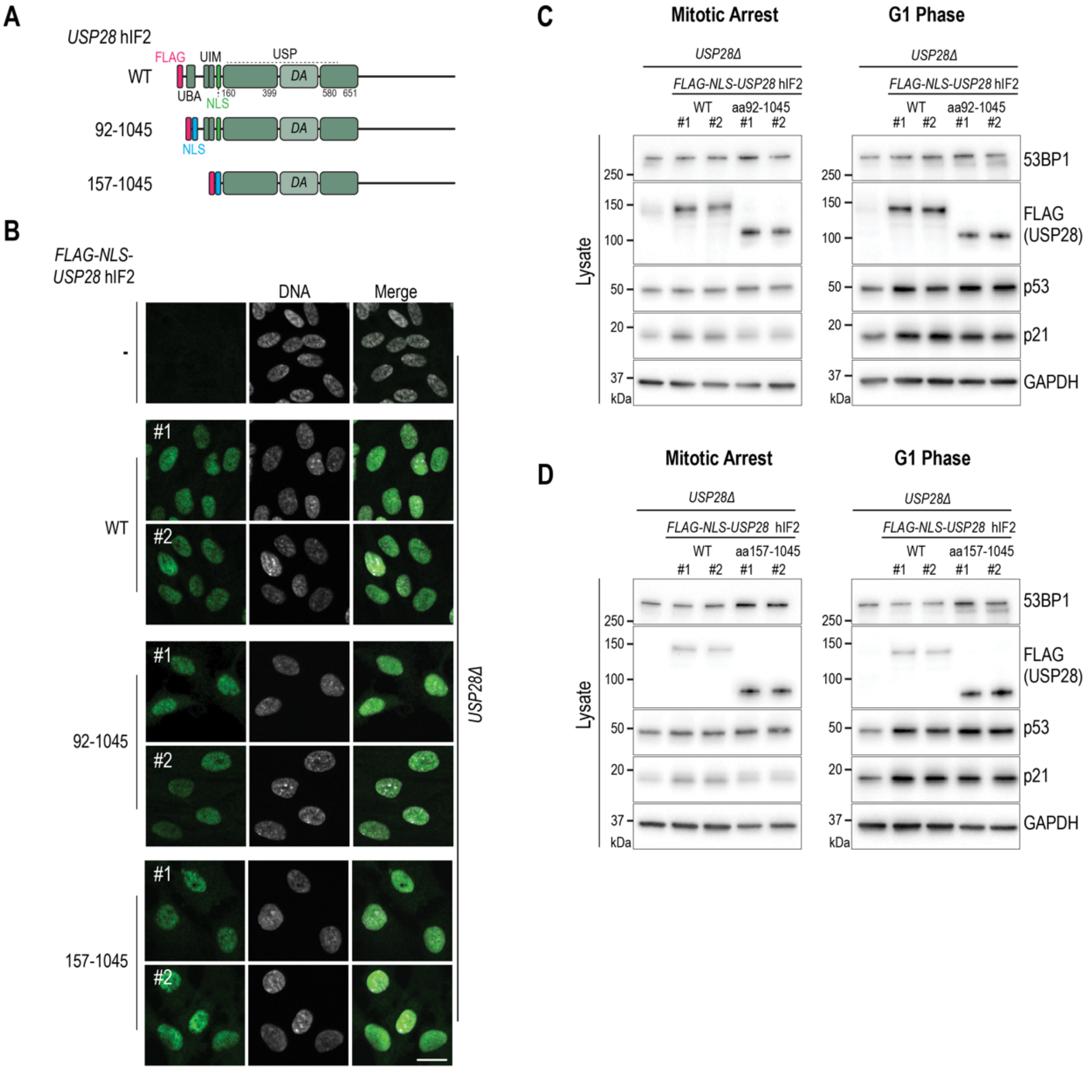
A
USP28 hIF2
FLAG
UIM
USP
WT
DA
UBA
NLS
160
399
580
651
92-1045
NLS
157-1045
B
FLAG-NLS-USP28 hIF2
DNA
Merge
-
WT
#1
#2
92-1045
157-1045
USP28Δ
C
Mitotic Arrest
G1 Phase
USP28Δ
FLAG-NLS-USP28 hIF2
WT
aa92-1045
#1
#2
Lysate
250
150
100
50
20
37
kDa
53BP1
FLAG
(USP28)
p53
p21
GAPDH
D
Mitotic Arrest
G1 Phase
USP28Δ
FLAG-NLS-USP28 hIF2
WT
aa157-1045
#1
#2

Figure S7: UBA and UIM are dispensable for the response to prolonged mitosis

(A) Schematic representation of the full-length USP28 (FL) and two N-terminally truncated fragments that lack the UBA (92-1045) or the UBA and UIM (157-1045). All transgenes are fused to a FLAG tag for comparison.

(B) Immunostaining of USP28 transgenes (depicted in A) expressed in *USP28*Δ RPE1 cells. Transgenes are fused to a FLAG tag and NLS from nucleoplasmin, enabling visualization and nuclear localization. Representative images of two independent single clones (#1 and #2) are shown for each transgene. Scale bar: 10 µm

(C-D) Immunoblot analyses of USP28 transgenes (depicted in A), assessing their ability to stabilize and activate p53 following release from prolonged mitotic arrest. Lysates were generated as described in Figure 2B. Lysates are soluble supernatants. GAPDH served as a loading control. The samples derive from the same experiment but different gels for 53BP1, p53, GAPDH, and another for Flag-USP28 were processed in parallel. Representative immunoblots from two independent experiments with similar results.
