# Supplementary material for: Cancer-associated USP28 missense mutations disrupt 53BP1 interaction and p53 stabilization
Source: Nat Commun. 2025 Dec 9;16:10310. doi: 10.1038/s41467-025-66341-3 (PMC12689633; doi:10.1038/s41467-025-66341-3)
Supplement: Supplementary file 2 — Reporting Summary [file 41467_2025_66341_MOESM2_ESM.pdf]

nature portfolio

Corresponding author(s): Franz Meitinger
Last updated by author(s): Sep 18, 2025

Reporting Summary

Nature Portfolio wishes to improve the reproducibility of the work that we publish. This form provides structure for consistency and transparency in reporting. For further information on Nature Portfolio policies, see our Editorial Policies and the Editorial Policy Checklist.

Statistics

For all statistical analyses, confirm that the following items are present in the figure legend, table legend, main text, or Methods section.

n/a	Confirmed	
☐	☒	The exact sample size (*n*) for each experimental group/condition, given as a discrete number and unit of measurement
☐	☒	A statement on whether measurements were taken from distinct samples or whether the same sample was measured repeatedly
☐	☒	The statistical test(s) used AND whether they are one- or two-sided<br>*Only common tests should be described solely by name; describe more complex techniques in the Methods section.*
☐	☒	A description of all covariates tested
☐	☒	A description of any assumptions or corrections, such as tests of normality and adjustment for multiple comparisons
☐	☒	A full description of the statistical parameters including central tendency (e.g. means) or other basic estimates (e.g. regression coefficient) AND variation (e.g. standard deviation) or associated estimates of uncertainty (e.g. confidence intervals)
☒	☐	For null hypothesis testing, the test statistic (e.g. *F*, *t*, *r*) with confidence intervals, effect sizes, degrees of freedom and *P* value noted<br>*Give P values as exact values whenever suitable.*
☒	☐	For Bayesian analysis, information on the choice of priors and Markov chain Monte Carlo settings
☒	☐	For hierarchical and complex designs, identification of the appropriate level for tests and full reporting of outcomes
☒	☐	Estimates of effect sizes (e.g. Cohen's *d*, Pearson's *r*), indicating how they were calculated

Our web collection on statistics for biologists contains articles on many of the points above.

Software and code

Policy information about availability of computer code

Data collection	Only commercial software was used. In particular: CQ1 Software (Yokogawa); Image Lab 6.1 (BioRad)
Data analysis	Only commercial software was used. In particular: FIJI, Excel, Prism, Cell Pathfinder Software (Yokogawa)

For manuscripts utilizing custom algorithms or software that are central to the research but not yet described in published literature, software must be made available to editors and reviewers. We strongly encourage code deposition in a community repository (e.g. GitHub). See the Nature Portfolio guidelines for submitting code & software for further information.

Data

Policy information about availability of data

All manuscripts must include a data availability statement. This statement should provide the following information, where applicable:

- Accession codes, unique identifiers, or web links for publicly available datasets
- A description of any restrictions on data availability
- For clinical datasets or third party data, please ensure that the statement adheres to our policy

Source data are provided with this paper.

Research involving human participants, their data, or biological material

Policy information about studies with human participants or human data. See also policy information about sex, gender (identity/presentation), and sexual orientation and race, ethnicity and racism.

Reporting on sex and gender	n/a
Reporting on race, ethnicity, or other socially relevant groupings	n/a
Population characteristics	n/a
Recruitment	n/a
Ethics oversight	n/a

Note that full information on the approval of the study protocol must also be provided in the manuscript.

Field-specific reporting

Please select the one below that is the best fit for your research. If you are not sure, read the appropriate sections before making your selection.

☒ Life sciences ☐ Behavioural & social sciences ☐ Ecological, evolutionary & environmental sciences

For a reference copy of the document with all sections, see nature.com/documents/nr-reporting-summary-flat.pdf

Life sciences study design

All studies must disclose on these points even when the disclosure is negative.

Sample size	Sample sizes are indicated in all of the figure legends. Sample sizes were always at or above the standard for this type of analysis and only differences well above the level of statistical significance are highlighted.
Data exclusions	No data was excluded.
Replication	The number of replications are described in the method section.
Randomization	No randomization was applied.
Blinding	Blinding was not used for any of the reported experiments.

Reporting for specific materials, systems and methods

We require information from authors about some types of materials, experimental systems and methods used in many studies. Here, indicate whether each material, system or method listed is relevant to your study. If you are not sure if a list item applies to your research, read the appropriate section before selecting a response.

Materials & experimental systems

n/a	Involved in the study	
☐	☒	Antibodies
☐	☒	Eukaryotic cell lines
☒	☐	Palaeontology and archaeology
☒	☐	Animals and other organisms
☒	☐	Clinical data
☒	☐	Dual use research of concern
☒	☐	Plants

Methods

n/a	Involved in the study	
☒	☐	ChIP-seq
☒	☐	Flow cytometry
☒	☐	MRI-based neuroimaging

Antibodies

Antibodies used	All antibodies, with their vendor, catalog number, RRID and working concentrations, are described in the methods section.
Validation	Antibodies for USP28, 53BP1, p53 and p21 were validated by immunoblotting and immunostaining using gene knockout mutants.

Validation	Other relevant antibodies for GAPDH, MYC, Histone H3.3 are established and broadly used antibodies. Antibody information including RRID are listed in the material and method section.

Eukaryotic cell lines

Policy information about cell lines and Sex and Gender in Research

Cell line source(s)	The source of all cell lines is listed in table S1, which was submitted with the article. Most cell lines were purchased from ATCC, JCRB or other common vendors unless otherwise noted.
Authentication	The majority of cell lines were obtained directly from the ATCC or JCRB and were not revalidated.
Mycoplasma contamination	All cell lines were tested regularly for mycoplasma contamination.
Commonly misidentified lines (See ICLAC register)	Not used for this study

Plants

Seed stocks	n/a
Novel plant genotypes	n/a
Authentication	n/a
