# Supplementary material for: Cancer-associated USP28 missense mutations disrupt 53BP1 interaction and p53 stabilization
Source: Nat Commun. 2025 Dec 9;16:10310. doi: 10.1038/s41467-025-66341-3 (PMC12689633; doi:10.1038/s41467-025-66341-3)
Supplement: Supplementary file 3 — Description of Additional Supplementary Files [file 41467_2025_66341_MOESM3_ESM.pdf]

Supplementary Data 1. List of cell lines used in this study, including their source, genotype, and relevant genetic modifications.

Supplementary Data 2. List of plasmids generated or used in this study, with details on construct design, insert sequence, and purpose.

Supplementary Data 3. List of primers used for RT–PCR analyses, including target genes, primer sequences, and expected amplicon sizes.
